# Incentivizing green building technology: A financial perspective on sustainable development in India

**DOI:** 10.12688/f1000research.154056.1

**Published:** 2024-08-15

**Authors:** Rakesh Kumar, Rajesh singh, Richa Goel, Tilottama Singh, Neeraj Priyadarshi, Bhekisipho Twala

**Affiliations:** 1 Uttaranchal Institute of Management, Uttaranchal University, Dehradun, Uttarakhand, India; 2 Department of Research and Innovation, Uttaranchal University, Dehradun, Uttarakhand, India; 3 Symbiosis Centre for Management Studies, Noida, Symbiosis International Deemed University, Pune, India; 4 Department of Electrical Engineering, JIS College of Engineering, Kolkata, 741235, India; 5 Digital Transformation Portfolio, Tshwane University of Technology, Staatsartillerie Road, Pretoria West, Pretoria West, 0183, South Africa

**Keywords:** Financial incentive, Green Building, Renewable energy, Technology, Government

## Abstract

Future viability depends on ensuring a sustainable society because green energy methods may efficiently reduce greenhouse gas emissions. Nevertheless, stakeholders, consumers, and developers continue to be notably ignorant of the financial incentives connected to green technology. Moreover, there is still a dearth of studies on the range of financial incentives offered by different authorities in India. Monetary incentives, such as tax breaks, indirect tax exemptions, and refunds, are crucial in encouraging the use of green technology in the modern world. This study explores the importance of financial incentives for green building technologies in India, which also looks at the wide range of incentives provided by federal, state, and local governments. Furthermore, the study highlights various state government programs such as goods subsidies, exemptions from local taxes, and fee waivers. Notably, several incentives aimed at consumers, developers, and other stakeholders have been implemented by the Indian Green Building Council (IGBC).

This review study emphasizes the effectiveness of financial incentives in green construction projects and draws attention to a clear knowledge gap regarding the adoption of green technology. This study also provides insights into potential future directions. Studies and research results emphasize the importance of spreading the word about financial incentives as a key factor in determining the adoption of green technologies. Many parties, including governmental organizations, municipal governments, developers, and clients engaged in green building technology projects, stand to gain increased awareness.

## 1. Introduction

Local governments can provide financial and technical incentives to support the creation of green buildings. Several Commonwealth nations have widely implemented financial incentives, notably property tax assessment incentives, for green construction. Property tax assessments for green buildings have been shown to stimulate green construction practices at the municipal level. ^1^ Technological advancements have resulted in the overexploitation of natural resources and environmental degradation. global primary energy consumption is anticipated to increase by 1.6% per year between 2009 and 2030. Green construction strives to lessen the environmental effects of buildings, while also creating a better environment in which to live and work. ^2^ The concept of green buildings has evolved for a sustainable society and efficient resources without an extra cost burden. Green buildings are life cycle projects that help build a sustainable nature. Green buildings use recycled materials, less water, and improve energy efficiency; thus, incentives are needed to stimulate adoption in the construction sector. There are limited traditional energy sources that would not be available for future society. ^3^ Green structures have fewer harmful effects on the environment while improving occupant health. The effects of buildings on the environment are frequently understood, and the perceived costs of green buildings are unknown. Green buildings can lead to life cycle savings of 20% of total construction costs more than ten times the initial investment. ^4^ For many building projects, both green buildings and traditional construction approaches are considered in the building design and construction. Several businesses and research organizations are working to quantify the advantages of green building construction. The difficulty lies in distinguishing between green building features and typical changes in company operations. ^5^ The building sector has considerable environmental and public health impact. Buildings account for more than 40% of total global CO2 emissions. Negative environmental, social, and economic repercussions can be mitigated using sustainable and green solutions. Green specifications and green building rules are common examples of green development in the construction sector. ^6^

**The objectives of the study are as under**:

1. To study and analyze green building concept in Indian landscape.
2. To study and analyze green building incentives and policies in the government and various authorities in India.
3. To study and analyze effectiveness of green building concept in sustainable development goal.

## 2. Review of literature

**Figure 1.**
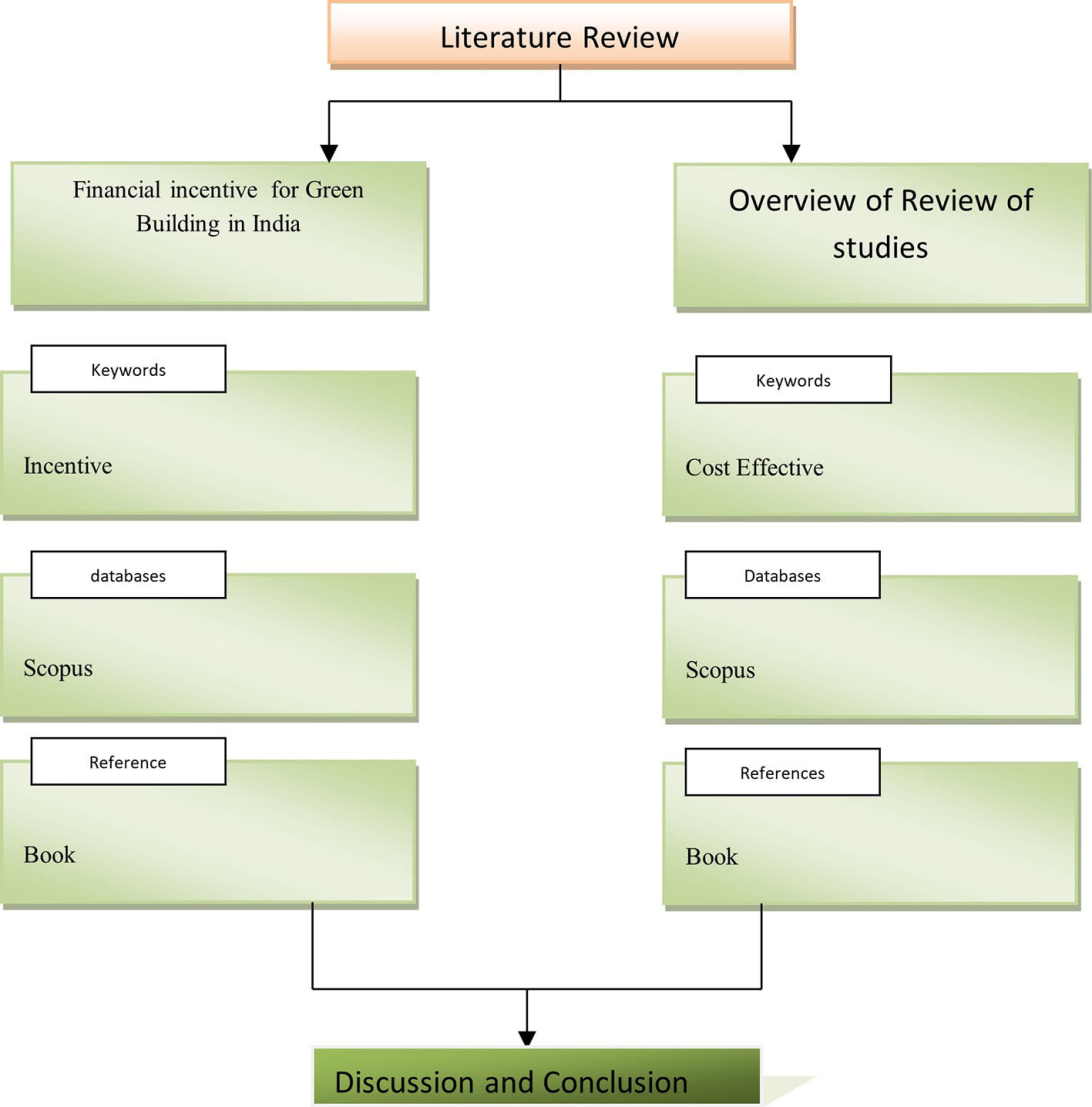
Systematic literature review.

The adoption of (Green Building Technologies) GBTs has several economic, social, and environmental advantages. There is a limit to traditional energy sources that are not cost-effective. The green building project provided renewable energy sources that saved energy up 24 %, cost effective, and sustainable for nature. To promote effective and efficient use of GBTs, appropriate strategies and policies must be developed to eliminate industrial hurdles. ^7^ Government engagement has been deemed ineffective for supporting the development of green buildings. Local governments are nonprofit organizations that provide services to the community. As part of their attempts to support green-building construction, local governments offer two types of incentives. ^8^ Developers of green buildings in Malaysia are given a green building index certification (GBI) if they meet the standards. Index defined on some basic criteria related to sustainable nature. The panel provides ratings for the green buildings. Tax exemption is provided based on the rating provided by the panel. ^9^ H1: Providing a quality environment in infrastructure enhances the adoption of greenhouse technology. Green buildings (GB) have emerged as a method to satisfy building demands while lowering energy usage during the 1970s and the 1980s. GBs may now be observed in significant urban areas throughout Vietnam, namely, in Hanoi and Ho Chi Minh City. In 2013, 41 GB projects were recognized and registered with seven distinct grading systems. ^10^ Numerous public initiatives have been implemented to encourage green development in the private sector. Green building regulations were classified into two groups in this study: regulatory and incentive-based regulations. Buildings that are newly constructed or renovated must fulfil the LEED or LEED-equivalent criteria according to regulatory rules. Green developers can obtain numerous tax credits, money, and funds using financial incentives. Green building developers can save time and money by expediting the construction permits and planning approval processes. Current financial incentives are seldom dispersed to private sector developers in several situations. This might be because they were unable to offset construction or repair expenditures owing to their greening properties. Figure 1 showing Process of comprehensive Literature reviews. Green building projects financed by different authorities at a reasonable cost. ^11^

## 3. Methods

The study was based on secondary data obtained from previous research, government websites, and departments. Study an analysis of existing incentives given by the Indian government and the respective state government. This article provides concrete examples of the use of documents in the research process, in addition to describing the types and characteristics of documents and outlining the benefits and constraints of document analysis. It describes how to use document analysis for a grounded theory study and provides a practical method for doing so. ^12^ This study uses specific areas from the Scopus and Web of Science databases. Specific keywords used for searching related documents. The search documents were limited to journal articles. Further peer-reviewed journals, books related to green technology, conference proceedings, and other documents related to green buildings were included in this study. In addition, many documents that are available with different maturities have been explored, such as municipalities, state governments, central governments, budget incentives, and NGT cases. Recently, India announced an incentive for green buildings in its budget proposals.

## 4. India’s green building efficiency landscape

It is generally known that consumer demand for energy-efficient devices is low. For instance, in India, biogas programmers supplied to rural households with considerable subsidies have not produced the desired outcomes. Given that energy expenses are within the reach of affordability, the limited awareness of urban families is also accompanied by limited motivation. More than four million people died in 2012 because of biomass-based fuels, which are still used by approximately three billion people. In developing nations, fuel stacking is a result of extreme variation between urban and rural areas, available fuels, and individual human preferences. The major obstacles are access to renewable energy and the availability of clean energy appliances. ^13^ Liquefied petroleum gas (LPG), electricity, urbanization, and lifestyle changes have caused a shift away from conventional fuels. If non-Kyoto emissions are considered, switching to LPG and electricity will have positive effects on both health and the environment. But not below the WHO recommendation, improved biomass cookstoves can reduce indoor exposure by percent to 86 percent. ^14^ This study examined home decision-making practices in Delhi, India, a market for energy-efficient lighting and appliances. This study examines the energy efficiency gap using an interdisciplinary framework of behavioral economics. It has been discovered that those who are more patient and less present-biased are more inclined to purchase energy-saving appliances. Time preferences are relevant for more expensive purchases, such as refrigerators, but they are less effective as an explanation for less expensive items like light bulbs. ^15^

The concept of the energy code was introduced in 2007. This is related to the interior system, outside electricity, and other services related to energy. Gujrat first introduced this concept. The model specifies the energy limit for specific purposes. For example, the energy of the hot water. It is also useful for a sustainable nature, as it reduces the emission of greenhouse gases. The Global Change Assessment Model (GCAM), which examines air pollution emissions and climate mitigation strategies, integrates energy, economics, land use, water usage, and climate systems. The global assessment models GCAM-India and GCAM-Gujarat included supplementary data for India and Gujarat, respectively. Green buildings have been created to improve tenant health while minimizing harmful environmental effects. Although the perceived costs of green buildings are unknown, the environmental effects of buildings are frequently underestimated. More than ten times the initial investment may be made via life cycle savings from green buildings of 20% of all construction expenditures. ^16^

**Figure 2.**
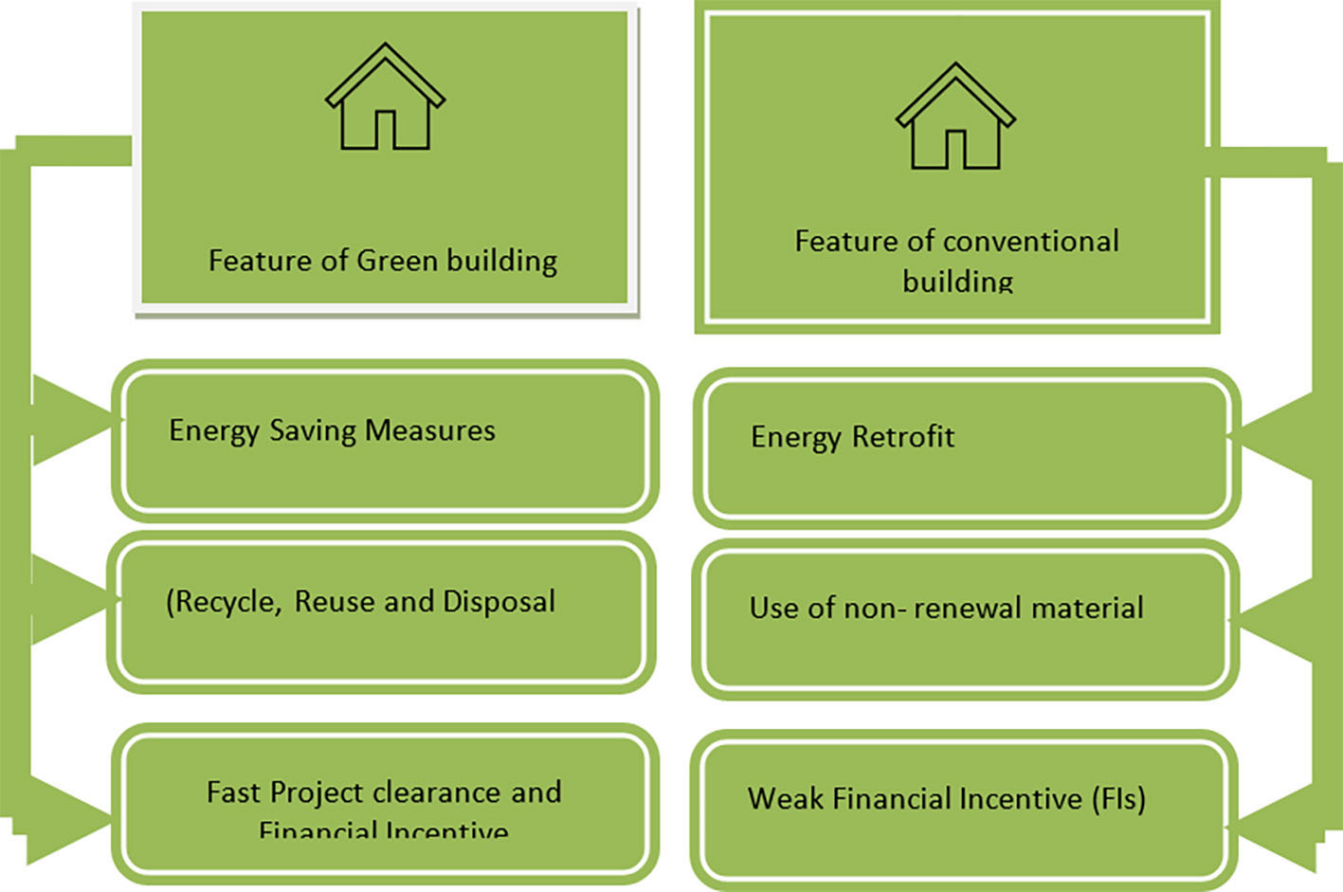
Energy landscape per in residential and commercial building.

India’s energy consumption is currently expected to be 600 MTOE, an increase of 50% between 2007 and 2017. According to the IEA India Report 2020, industrial establishments consume the most (42%), followed by the residential, transportation, and service sectors (see Figure 2). Industries consume 44% of the electricity, followed by households (24%), agriculture (18%), and commercial buildings (8%). ^17^

It is impossible to overstate the significance of energy as a driving force behind economic growth. With the UN’s SE4ALL declaration, the importance of energy in the development process has been acknowledged worldwide. Due to the Government of India’s key programs, such as Sau Bhagya and PMUY, India has seen remarkable advances in the provision of energy services, both in terms of access to power and the availability of cooking energy. India has developed into a lively, fast-growing economy with a rapidly changing socioeconomic profile that has resulted in the emergence of a middle class and a higher proportion of people living in cities. The literature suggests that this might increase the purchase of energy-intensive assets. According to estimates, if half of Indians purchased one refrigerator, the country’s GDP would increase by 10%. This study proposes a new method for measuring energy poverty in India based on a solid collection of measurements comprising several aspects. Given that India’s socioeconomic profile is changing, it is important to build a thorough understanding of the problem of energy poverty, considering a larger collection of variables encompassing both access and cost features. ^18^

### 4.1 Financial incentive for green innovative building in India

It is predicted that between 2010 and 2020, global energy consumption will increase by 106 billion Btu. This indicates a strong 56% increase in energy use over the following 30 years. As economic expansion is the goal, emerging nations will see 85% of this gain. Increasing the energy efficiency of the building sector has several advantages including improved internal and external air quality. One of the best methods to improve a nation’s energy supply security and reduce carbon emissions is to increase building energy efficiency. ^19^^,^^20^ Italian towns have been essential participants in the adoption of building energy regulating rules (BERC) in order to decrease the environmental effect of newly built and refurbished structures. ^21^ The journals used for this research are listed in Table 1.

**Table 1.** Types financial incentive for green building in India.

Type	Description	Ref
Subsidies and grants	Green buildings (GBTs) are advantageous in terms of energy use and emissions, low maintenance and operating costs, and enhancing productivity and health. GBTs are created for a longer period, are cost-effective, and use the least number of natural resources.	^22^
Loan and tax incentive	Customers who purchase homes in environmentally friendly developments that lower carbon emissions and support renewable energy can apply for a green housing loan.	^22^
IGBC	The green building attributes have been benchmarked using a Data Envelopment Analysis (DEA). For benchmarking reasons, buildings in several climatic regions were selected, including hot and dry, warm and humid, and composite climates. Utilization of BIS-recommended waste products, a rise in environmental consciousness, and specialized facilities for service workers were also noted.	^22^^,^^23^
Green Banking	A growing concept, green banking is important for addressing climate change, operating the capital markets, and fostering sustainable economic growth. Triodos Bank, a Dutch bank, initially launched GB in 1980. The State of Florida then implemented GB in 2009. GB is useful since it fosters banks' goodwill and brand image and shows their commitment to environmental conservation.	^22^

Green building incentives are crucial for the advancement of green construction. However, there has not yet been a thorough examination of the subject’s body of knowledge. Through a systematic study, this research seeks to identify recurring patterns in studies on incentives for green construction. Additionally, it reveals the necessity for the government to change its strategy to encourage owners to undertake green-building development. ^24^ It is generally acknowledged that the building sector has negative effects on the economy, society, and the environment. Green buildings have been recognized as an effective substitute for traditional buildings to reduce or completely eliminate the harmful effects of construction operations. The adoption of green building technology (GBTs) has several important sustainability advantages. The adoption of GBTs is not free of obstacles or difficulties. Extensive research has been conducted on obstacles to the adoption of GBTs and practices. Meanwhile, few studies have examined these in emerging nations. ^25^ Different types of technology and advanced equipment in computers are used to represent green product innovation, which is a method of production that prioritizes sustainability. Because depending solely on market forces makes it impossible to accomplish quick development, proper laws and rules are required that can be effective for green projects. This study examined the impact of tax incentives for R&D and environmental legislation on the development of green products. The creation of items that are simple to recycle, use, and regenerate after use is known as “green product innovation.” The technology used for information and communication (ICT) is a classic example of green product innovation. Technology can assist different fields by increasing their effectiveness. Research and innovation are important factors for green-building projects that can find grey areas that can be improved. Government agencies have been obliged to impose restrictions on polluters through legislation and environmental regulations because of the ongoing degradation of the environment since the 1970s. The “blue sky defense battle” is a three-year action plan that was released by the Chinese State Council in June 2018 to encourage energy efficiency and emission reduction in significant industries. Environmental legislation has a considerable positive impact on businesses’ “green” conscience. Innovation in green products begins with R&D, and the product is launched into the market. This involves several closely related activities with high tax sensitivity. Environmental regulation is the most prevalent type of punishment. With its rapid economic expansion, low per capita income, lax legal system, and ineffective tax structure, China is an emerging nation. Table 1 showing different types of financial incentive for green building. This study focuses on the analysis of the effects of environmental legislation and R&D tax policies on the development of green products, which is of major innovation relevance. ^26^

**4.1.1 Subsidies**

The impact on the environment is receiving more attention as there is a growing need for the discovery and exploitation of natural resources. Regulating this through governmental organizations and legal standards is one approach. Several environmental and green building rating methods have recently been developed to aid in the design of green buildings. ^20^ Various levels of government worldwide are focusing on the growth of green buildings. Local governments offer two primary types of incentives to promote green buildings. The impact of property tax assessment incentive models on local examples of green buildings has not been studied thoroughly. ^27^ Green finance is defined as the provision of funding for a range of environmentally beneficial initiatives including waste management, recycling, alternative energy, energy efficiency, renewable energy, and the growth of green industries. The European Commission defines green finance in financial services as an investment choice that considers governance and social and environmental factors. A broad spectrum of policy tools are referred to as “market-based environmental policy instruments” (MBIs). Their shared traits are the use of market power and rivalry to accomplish environmental goals. The categories of market-based instruments for green buildings in India are listed in Table 2.

**Table 2.** Categories of Market based instruments for green building in India.

Category	Description	Instrument	Reference
Price- Based instrument	These included tax rebate, subsidy and other incentive which reduce from cost of product. It also gives long-term benefits.	Subsidies, Tax rebate, Loan, Grant etc.	^27^
Right Based Instrument	This incentive proved based on quantity. This led to more trade. It means more trading, more incentive. This type of incentive is provided at builder level.	Quota Carbon incentive Lead Trading	^22^^,^^27^
Market Friction reduction Based instrument	These are legislative tools designed to enhance market efficiency through better information or rising consumer demand. Non-financial MBI is this kind. Examples of this include the labelling of energy-efficient products, peer-to-peer networking occasions, parking spaces for electric vehicles, and environmentally friendly public procurement.	Insurance scheme Debt scheme Leverage Products differentiate	^27^

**Figure 3.**
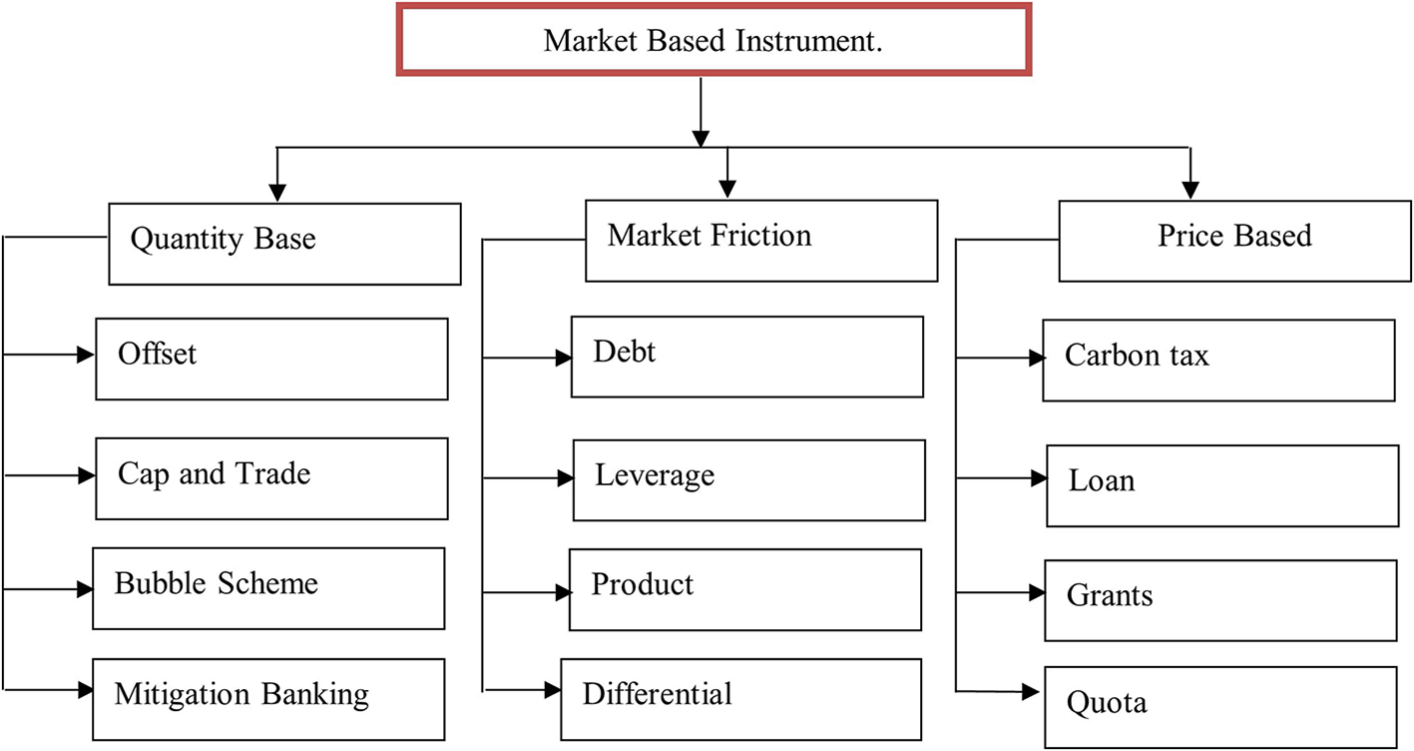
Market based instrument.

Figure 3 shows the market-based instrument, which is divided into three parts: quantity-based, market friction-based, and price-based. Quantity-based instruments include banking incentives, offsets, and schemes related to quantity. Market friction instruments include debt, leverage, differentials, and products. Price-based instruments include taxes, loans, grants, and quotas.

**4.1.2 Loan**

Buildings have a significant impact on climate change; however, they can help planners and environmentalists by decreasing greenhouse gas emissions. However, several barriers have made it difficult to switch to green buildings. Energy saving is a significant challenge for society. The government provides incentives based on energy savings and reduction in greenhouse gas emissions. ^28^ Both the natural monopoly and appropriability failure components result in a two-fold market failure. The shortcomings of public sector banks cause financial market difficulties. All PPPs must internalize the risk of interest rate changes according to the policy. To prevent the state from being forced to bear the consequences, it must restrict the parameters under which renegotiation is conceivable. ^29^ The loan facilities for green buildings in India are listed in Table 3.

**Table 3.** Loan facility for green building in India.

Description	Loan facilities
Special Feature	Discount on margin money– 5 percent Interest concession-.25% Processing Fee- Waive off
Term	25 years up to 70 years of age
Quantum of loan	Min 5 lac
Rate	Fix and floating
Documentation	Income proof along with tax return

Sources: Prepared by author from different bank rate.

**4.1.3 Tax incentive**

In green buildings, these resources are conserved while being used as efficiently as possible. Actors who spend money and investors receive distinct benefits; hence, there are split incentives in this industry. Quantifying the value of investments in green buildings can be difficult local governments in India have granted a 50% building tax discount and there is rebate in property tax for projects that receive (Indian Green Building Council) IGBC greenhouse certification. India has promised to reduce its carbon emissions by 80% by the year 2050 from 2005 levels, a reduction of 35% from those levels by 2030. Building energy efficiency has the highest potential for energy saving at the lowest cost. This study examines current energy usage patterns in India’s home construction industry. It provides a general overview of the numerous obstacles and difficulties that arise when implementing energy efficiency programs in homes. ^28^

**4.1.4 IGBC**

Green building rating systems are frameworks that have been developed to assess and certify the environmental performance of buildings. These rating systems evaluate various aspects of a building’s design, construction, operation, and maintenance to promote sustainable and environment-friendly practices. They provide a standardized approach for measuring and recognizing the green attributes of buildings. These rating systems provide guidelines, benchmarks, and certifications to encourage sustainable building practices, reduce environmental impacts, and enhance occupant well-being. They play a crucial role in driving the adoption of green building practices, and are often used as benchmarks for sustainable construction projects. ^30^

“To make the built environment sustainable for everyone and to be a global leader in sustainable building by 2025,” reads the organization’s mission statement. The organization’s signature yearly event focused on environment-friendly structures is the Green Building Congress. An integrated method called “green building rating” considers the effects of the resources consumed across life cycles. It employs traditional architectural techniques along with cutting-edge technical advancements in design inspired by the five elements of nature (Panchabhutas). The IGBC rating program is now available for all five climatic regions of India and is National by Choice, as well as Global by Performance. ^31^ Various state government schemes for green-building projects in India are listed in Table 4.

**Table 4.** various state government scheme for green building project in India.

State	Department	Free floor area ratio	Reference
Punjab	Department of local Government (Town Planning)	Five percent	( https://igbc.in/igbc/)
	Department of housing and urban	Ten percent	( https://igbc.in/igbc/)
Rajasthan	Urban Development	.75 for gold and .75 for platinum	( https://igbc.in/igbc/)
	Rajasthan Investment Scheme	-	( https://igbc.in/igbc/)
West Bengal	Municipal Department	Ten percent	( https://igbc.in/igbc/)
	Development Authority	10 percent for FAR	( https://igbc.in/igbc/)
UP	Housing and urban planning department	Five percent for gold	( https://igbc.in/igbc/)
	NOIDA	Fifty five percent for gold	( https://igbc.in/igbc/)
Andhra Pradesh (AP)	Industries and commerce department	-	( https://igbc.in/igbc/)
	Municipal Administration	20% Reduction on Permit Fees	( https://igbc.in/igbc/)
Himachal Pradesh	Town and country planning Department	Extra 10% for Gold/Platinum rating by IGBC.	( https://igbc.in/igbc/)
Jharkhand	Urban Development and housing Department	Up to 7 percent	( https://igbc.in/igbc/)
Haryana	Town and country planning Department	9%,12% and 15% for silver, gold and Platinum respectively	( https://igbc.in/igbc/)

**4.1.5 Rating system under IGBC**

The IGBC is a leading organization in India that promotes sustainable practices in the built environment. IGBC has developed several rating systems to assess and certify green buildings. The major rating systems offered by the IGBC are as follows:

• Green Homes: This rating system is based on efficient energy, water, and electricity systems, and cost-effective equipment at home. Green home rating system based on the interior house system
• Green New Buildings: This rating system assesses water, energy, electricity, construction, material used, and type of living. This is only applicable to new buildings.
• Green Existing Buildings: This rating system focuses on the operational aspects of existing buildings. This helps building owners and operators improve the environmental performance of their structures in areas such as energy conservation, water management, waste management, indoor environmental quality, and maintenance practices.
• Green Factory Buildings: This rating system was specifically tailored to industrial buildings and manufacturing facilities. It evaluates energy conservation, water efficiency, site selection, material selection, indoor environmental quality, and waste management practices.
• Green Schools: This rating system assesses the environmental performance of educational institutions, including schools and colleges. It focuses on categories such as site selection, water and energy efficiency, indoor environmental quality, transportation, and innovation.
• Green Cities: The IGBC Green Cities Rating System is aimed at developing sustainable and smart cities. It covers various aspects including urban planning, governance, buildings, energy, water management, waste management, mobility, and social infrastructure.

**Figure 4.**
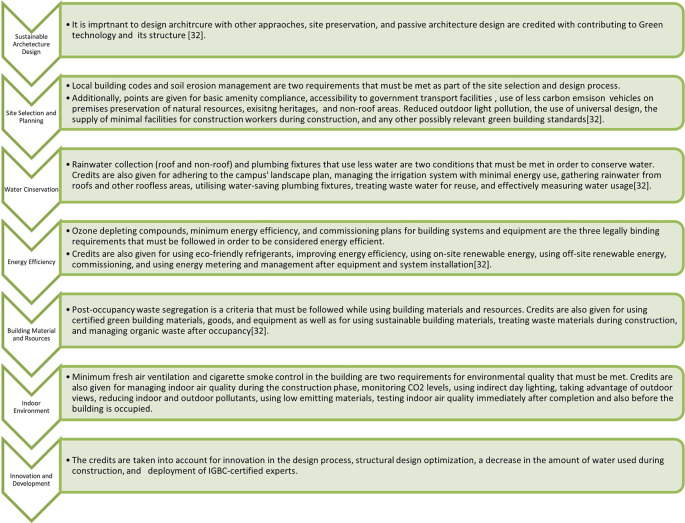
IGBC rating system parameter.

The construction industry has several negative environmental effects. People have become aware of the negative impacts of construction operations by nonprofit organizations. Figure 4 showing rating parameters. The two green building rating systems for existing buildings in India were compared in this study in terms of their water and energy usage. ^32^

**4.1.6 Central government and state government FIs for green building project**

Incentives are based on a rating system. However, state governments provide different incentives for different projects. Table 6 describes the different ministries that have given invtives for green projects. environment ministry provides fat-track GBs clearance. Urban departments incur additional costs. The municipal department provides 15 countries, and the Confederation of Indian Industry supports IGBC, which adheres to LEED global standards. Meanwhile, GRIHA is being specifically developed in accordance with Indian conditions with the Ministry of New and Renewable Energy funding. A national green building rating system for structures in India is called Green Rating for Integrated Habitat Assessment (GRIHA). It was created by TERI (The Energy and Resources Institute) with assistance from the Ministry of New and Renewable Energy of India. The five “R”s of sustainable development—Refuse, Reduce, Recycling, Reuse, Recycle, and Reinvent—are adopted by GRIHA. ^33^ The financial incentives provided by different governments are given in Table 5.

**Table 5.** FIs for green building project by central government.

	Type of incentive	Reference
Ministry of environment, forest and climate change	Provide fast track environment clearance for GBs	( https://igbc.in/igbc/)
Ministry of urban development	Extra ground coverage cost from 1% to 5%	( https://igbc.in/igbc/)
Municipal corporation	15% tax discount	( https://igbc.in/igbc/)
Small industrial development bank of India	All green projects will be eligible for financial assistant at discount rate	( https://igbc.in/igbc/)

**Table 6.** Ex ante, post ante FIs for green building.

Evaluation	Approach	Method	Advantage	Disadvantage	Ref
Ex-Post	Green Rating for Integrated Habitant Assessment (GRIHA)	The National Advisory Committee (NAC) was established to oversee the national rollout of GRIHA. High-ranking representatives from numerous government agencies are members of the NAC.	Quality of product will no be compromise	Number of compliance require	^27^
Ex Ante	Forecasting (Energy Economic Model)	In comparison to their developed counterparts, the bulk of Asian economies still have a long way to go in terms of their building energy efficiency policies. Green building standards are widely accepted and strictly adhered to in China, Singapore, Taiwan, South Korea, Hong Kong, and Japan. Standards are created in India; however implementation strategies require improvement. Several model developed for green building like Solar water heating system (SWH) and Green building sustainability model.	Good energy sources for cost and within requires time framework. SWH is low cost appliance and sustainable product for household.	It is useable in sun light within area where direct sun rays available. Initial cost is high which cannot afford by low income class people.	^20^
	Back casting	A business jet is a crucial step in a green project. The back casting method aids with this planning. Instead of doing activities that are essentially just the continuation of current techniques into the future, future desired situations are imagined, and then processes are specified to reach those conditions. To put it simply, back casting is the reverse of prediction.	Benefit for consumer perspective	Future risk involved	^27^

## 5. Productivity of financial incentive for GBs in India

**Figure 5.**
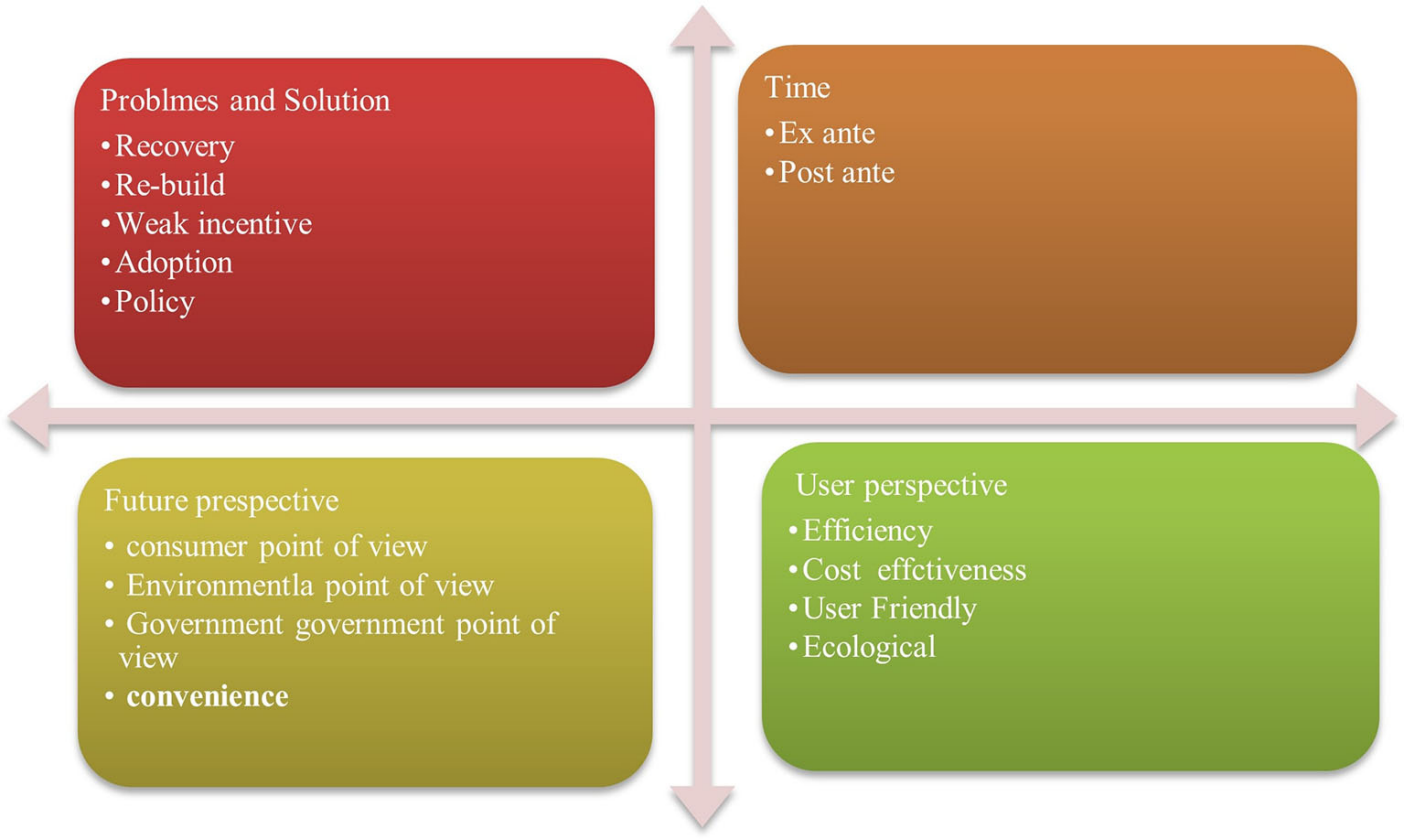
Evaluation Productivity of FI for Green building through review.

13.2% of the worldwide gross domestic product is accounted for by the building industry (GDP). Although adopting sustainable building practices is sluggish owing to obstacles, they can lessen the negative effects of the construction industry. Figure 5 showing evaluation productivity of financial institutions (Fis) Project schedule extensions, a dearth of research and development efforts, and lax enforcement of construction codes are obstacles specific to India and a few other developing nations. ^34^ The goal of this study is to identify the most significant locations, organizations, authors, journals, keywords, and references in the literature on stakeholder studies of green buildings from 2007 to 2021. AHP, “safety risk,” “evolutionary game,” “occupant satisfaction,” “rehabilitation,” and “green apartments” green apartments are among the knowledge base areas of greater emphasis, according to a cluster analysis. ^35^

### 5.1 Time evaluation

With regard to ex ante, age, hedonic characteristics, rent-free periods, renovation, and amenities of a building are projected to have a large and beneficial impact on prices, with younger, taller, and taller structures commanding higher rental rates. Buildings that have been in the market for a longer time may likewise command higher prices, which would indicate greater rental stability and less fluctuation in the tenant’s credit rating. The location and type of investors (principals, agents, or institutional investors) – private developers, REITs, real estate investment managers, and municipal government investors – impact the value of a green building in central London. To calculate the average locational value of buildings in a neighborhood, we used postcodes and transportation networks. Using data mining and decision tree techniques, this study aimed to identify useful models and trends. Four HSEE management fields (health, safety, environment, and energy), as well as four structural, architectural, mechanical, and electrical installations were used as variables. From the viewpoint of safe green buildings, construction competency was treated as a dependent binary variable. ^36^ For ex-ante, two variable selections, forecasting and back casting ( Table 5).

### 5.2 User perspective

Energy efficiency is a term used to define several requirements that must be met in green construction. This includes utilizing energy-efficient machinery, climate-appropriate materials, and services and facilities that are necessary for the building’s intended purpose. In addition, the energy embodied in building construction and demolition should be considered. The social, Environmental, and Economic (SE2) dimensions are three key factors that should be considered when developing green buildings. The GBRS aids in assessing the performance of the criteria in buildings that have an impact on society, such as waste output, energy consumption, and indoor air quality. The establishment of standards for the creation of rating systems and issues with the evaluation and validation of such systems are the primary focus of research on green building rating systems. In this study, rather than using exact quantitative analysis, a fuzzy inference system with decision-making rules was employed to mimic the qualitative components of human knowledge and reasoning. ^37^ The term “green” refers to a perspective that is acceptable and resourceful while discussing economic and environmental concerns. Natural resources and environmental services can be included in national revenue and wealth accounting, thanks to green infrastructure. This might cause the traditional infrastructure’s single-profit purpose to change into a multi-profit one as a dynamic social system. The focus of attention on the planet is on infrastructure and how it affects sustainability, social cohesion, and economic development. By considering the network linkages between the GI and GE indicators, this study classified and rated GI criteria. Analyses were performed to determine how the GI-ranked criteria affected GE implementation. The goal of this research was to examine how GI criteria and GE enactment in an SD scenario interact. ^38^ As of January 31, 2006, the total installed capacity of electric power-generating facilities operated by utilities in India was reported to be 123,901 MW. Between April 2005 and January 2006, the country’s peak electricity demand (10,556 MW) and energy supply (41,630 million kWh) were approximately 11.6% and 8.0%, respectively. The Indian government planned to electrify all homes in 2012 and the final villages by 2007. The lowest levelized unit cost of electricity (LUCE) of all user options was provided by biomass gasifier-based power plants (BGPP). The annual amount of electricity delivered and the project’s total annualized cost directly affect the LUCE provided by BGPP. ^39^

### 5.3 Potential perspectives

Buildings account for 40% of all energy usage and have substantial environmental, social, and economic impacts on the sector. Additionally, buildings produce greenhouse gases, with 43% of their waste going to landfill. Assessment methods have been developed to support the construction of green buildings, and the World Green Building Council has coordinated these efforts on a worldwide scale. These resources provide credit for several sustainability topics. Many countries are providing incentives for green-building projects. This is not only good for a sustainable nature but also cost effective.

**5.3.1 Consumer perspective**

Green buildings are among the best ways to promote environmental sustainability. People believe that green buildings’ greater price premiums are justified by their environmental advantages. We created a framework based on the Howard-Sheth model of customer behavior. The next step was to create a pricing model to estimate the price premiums of green buildings using an artificial neural network. Green construction of home items has become increasingly popular. However, will customers accept the product? Are they prepared to spend (WTP) more money? Do consumers’ perceptions, attitudes, intentions to buy, and WTP remain stable over time? This essay is based on a survey of homebuyers in Kaohsiung City, Taiwan. ^32^

### 5.4 Problems and solutions

The building and construction sector has had a tremendous negative impact on the environment, society, economy, and human health. 30% of the world’s greenhouse gas emissions and 40%–50% of water pollution are produced by the building industry. Table 7 showing problems and solutions. Green building (GB) construction aims to address these problems by increasing the energy efficiency and reducing the negative effects of construction. The development of GB in the US is hindered by three key issues. Some individuals believe that GBs have fallen short of their promises, such as the realization of energy conservation. It is quite difficult to convince people to obtain GB at a higher cost. It is crucial to increase the knowledge of the meaning of GBs among architects and designers. The accomplishment of these objectives is accompanied by significant hurdles. One of the biggest problems is that the laws governing the actual building process and the NZEB, CNB, and GBs are not well understood. However, widespread adoption may have issues. Instilling a feeling of accountability is crucial to sustainable development.

**Table 7.** Problems and solutions.

Description	Issues	Solution	Ref
Financial Inactive	Owners of green buildings in Indonesia urge banks to assist their initiatives more. Only Bank Jawa dan Banten (BJB) and Bank Negara Indonesia (BNI) have offered green loans thus far. Owners claim that the existing regulation's regulations and method need to be explained in more depth. According to 40% of respondents, the slow progress of green building in Indonesia has been caused by building tenants' lack of awareness. In Indonesia, there wasn't enough data from studies and case studies to demonstrate the benefits of going green and how quickly they would pay for themselves.	Awareness program shall be launch for developer as well as consumer related to FIs in green building and green technology.	^34^^–^^41^
Cost	In Ghana, cost is regarded as a significant and delicate obstacle to the adoption of GBTs. It is common knowledge that GBTs are considerably more expensive than their conventional counterparts. The increased adoption fees for GBTs can significantly impede their adoption in Ghana, a developing nation where poverty is pervasive. Incentives can help Ghanaian builders overcome the financial barrier preventing them from implementing green building techniques (GBTs). It is envisaged that practitioners would be able to manage the costs related to the deployment of GBTs as they gain more experience. Because it can lower overall efficiency, GBT performance uncertainty can also be fatal to a green building project.	Rebate, exemption and cost effective policy shall launch for encouraging of green building technology.	^42^^,^^43^
Policy Support	An external constraint, such as a poor commissioning facility, has a detrimental effect on stakeholder interests; tiny markets limit the use of green grading systems, which makes premium and resale prices unappealing.	Fast clearance for developer and continuing benefit to consumers.	^28^

## 6. Effectiveness of FIs in criteria for green building site

Buildings are becoming increasingly necessary as the population increases. By 2020, it may be possible to reduce GHG emissions by 142 megatons (Mt) annually. Approximately 5 billion Asians, or 60% of the world’s population, are expected to live in these structures by 2030. Lack of knowledge and false perceptions are the two main obstacles to promoting the growth of green buildings. Table 8 showing effectiveness of Fis criterias. Green buildings in India are said to be more expensive and take longer to develop. Consequently, the inexpensive housing market was excluded because green buildings are considered a premium market. ^48^

**Table 8.** Effectiveness of FIs in criteria for green building site.

Criteria	Approach	Ref
Sustainable site	Based on a careful examination of site planning and sustainability principles, the Urban Renewal Agency (URA) has established a theoretical framework for land-use choices in urban renewal projects. The development of URA's plans is illustrated by the framework using three real-world case studies. A major emphasis is placed on the environment, and market-based economic systems are regarded as sustainable. A fundamental shift in economics has been brought about by ecological economics, and urban regeneration today include restoring and revitalizing older communities rather than merely eradicating large-scale slums.	^44^
Renewal Energy	Techniques for evaluating renewable energy in green building and neighborhood grading systems are used to promote the construction of green buildings. The present utility system is facing difficulties as a result of rising consumer demands, the depletion of fossil fuels, and decreased generation efficiency. To minimise carbon emissions, government rules encourage the use of alternative energy sources. Power electronic inverters are to be replaced with an energy conversion system based on synchronous generators and DC motors.	^45^^,^^46^
Durable material	Buildings in Greece should give top priority to initiatives that conserve water, reduce waste, improve indoor air quality, and use renewable energy. To minimise sprawl and maintain natural lands, water, and air quality, smart development, an urban planning and transportation concept, places a focus on compact, walkable regions. Locals and communities are active in development and smart growth efforts.	^47^
Water Efficiency	Sustainable sit helped to reduced water waste and recycling of water process which can be useful for same society. Green technology Avoid water wastage 30 to 50 %.	^47^
Waste management	Green technology manage waste efficiency which can be recycle.	^47^
Developers	Many people believe that environmentally friendly developments take more time and pay off less monetarily. However, properly-executed green developments perform incredibly well financially since they have increased marketability, reduced running costs, and better health and productivity for the populace. From the very beginning of operation, the benefits include a decrease in the need for water and energy. The water savings are between 30 and 50%, and the energy reductions are between 20 and 30%. Other advantages are: • Efficient maintenance charge • Lees dependency on resources • Waste management. • Marketing avenue • Fast clearance	^47^

## 7. Conclusion and recommendation

### 7.1 Conclusion

Detailed review of the financial incentives available for green buildings in India. The study examines subsidies, tax exemptions, rebates, loans, and other incentives provided by central and state governments. In addition, local state government authorities provide different incentives to encourage green buildings. This study identified the benefits of green buildings from a future perspective. The study revealed barriers in green building projects. studied the role of IGBC in green-building projects in terms of awareness, planning, and financial incentives. Technology implementation and policy support are important factors in green building project. This study finds ex ante and post ante analyses of green buildings. This study included all studies conducted in India and abroad to compare the growth of green buildings. There have been limited studies conducted on green building projects in India.

### 7.2 Recommendation

Sustainable energy with cost-effective prospects and reduction of greenhouse gas emissions is a significant challenge in our society. The green building concept is a one-point solution for green energy, efficient energy, the reduction of greenhouse gases, and durable products. However, to increase people’s trust, it is important to provide incentives and support for green building projects. This study makes the following recommendations for green building projects.

• Presently, there are many incentives provided by the government, such as subsidies and financing for projects. but the tax incentive is a gray area. The government provides tax incentives for projects as well as for consumers.
• There is always a challenge for new projects in terms of cost and trust. It is important to provide cost-effective and durable equipment that provides value added in the future rather than a reduction in cost. This enhances consumers’ trust of consumers towards green projects.
• Recycling of products is a basic requirement for a sustainable society. Green building projects should be emphasized towards the recycling of products, as they can be easily used in the same society. This study finds existing financial incentives for projects that are limited to the builder level. There should be more incentives for consumers, so that society moves towards green-building projects.
• Awareness is an important factor in green building projects. It is important to make people aware of green building projects.

### 7.3 Policy implication

Policy implications are related to green building growth with financial incentives and other benefits so that maximum outcomes can be achieved in the future. Furthermore, this recommendation can assist in green building project planning.

• The central government, as well as the state government-level energy incentive program, shall be introduced to reduce greenhouse emissions and use renewable energy sources such as solar energy and wind energy.
  ○ State governments should encourage the adoption of renewable energy sources at the local level. This can be achieved through more financial incentives than existing energy sources.
  ○ Financial incentives shall be given for green building materials, construction services, tax rebates, and future cost surety. People’s awareness programs play an important role in the adoption of green buildings.
  ○ More incentive shall be given to the renovation of existing source so that people can easily adopt new green building technology from existing technology.
• From a developer’s point of view, speedy and timely clearance played a significant role in the smooth implementation of green building projects. The government should introduce a speed-and-transparency project for stakeholders. In this way, people will be more attracted to investing money in green projects.
• From a consumer perspective, it is important to launch awareness programs about the financial incentives available for green energy as well as green building technology. People are not ready to adopt green building technology because of a lack of awareness about financial incentives.

## Data Availability

No data collected for this study.
