# Supplementary material for: Incentivizing green building technology: A financial perspective on sustainable development in India
Source: F1000Res. 2024 Sep 16;13:924. Originally published 2024 Aug 15. [Version 2] doi: 10.12688/f1000research.154056.2 (PMC11393528; doi:10.12688/f1000research.154056.2)
Supplement: Peer review history

# Reviewer report: Reviewer response for version 2

**DOI:** 10.5256/f1000research.171777.r324076

**Published:** 2024-09-23

**Authors:** Madhusmita Mohanty

**Affiliations:** 1 VIT-AP School of Business,, VIT-AP University Amaravati, Amaravati, Andhra Pradesh, India

Authors have incorporated all the queries. Hence I approved the article for indexing.

Is the review written in accessible language?

Yes

Are all factual statements correct and adequately supported by citations?

Yes

Are the conclusions drawn appropriate in the context of the current research literature?

Yes

Is the topic of the review discussed comprehensively in the context of the current literature?

Yes

Reviewer Expertise:

Finance, Banking

I confirm that I have read this submission and believe that I have an appropriate level of expertise to confirm that it is of an acceptable scientific standard.

# Reviewer report: Reviewer response for version 2

**DOI:** 10.5256/f1000research.171777.r324075

**Published:** 2024-09-18

**Authors:** Mohammed Ismail Iqbal

**Affiliations:** 1 College of Engineering and Technology,, University of technology and sciences, Nizwa,, Oman

Now the paper is updated and can be indexed.

Is the review written in accessible language?

Yes

Are all factual statements correct and adequately supported by citations?

Yes

Are the conclusions drawn appropriate in the context of the current research literature?

Yes

Is the topic of the review discussed comprehensively in the context of the current literature?

Yes

Reviewer Expertise:

Environmental Engineering, Sustainability,......

I confirm that I have read this submission and believe that I have an appropriate level of expertise to confirm that it is of an acceptable scientific standard.

# Reviewer report: Reviewer response for version 2

**DOI:** 10.5256/f1000research.171777.r324074

**Published:** 2024-09-17

**Authors:** Vinay Kandpal

**Affiliations:** 1 Graphic Era Deemed to be University, Dehradun, Uttarakhand, India

As authors have incorporated all the revisions suggested by reviewers, so I would suggest for acceptance of paper.

 Nice work done by Authors.

Is the review written in accessible language?

Yes

Are all factual statements correct and adequately supported by citations?

Yes

Are the conclusions drawn appropriate in the context of the current research literature?

Yes

Is the topic of the review discussed comprehensively in the context of the current literature?

Yes

Reviewer Expertise:

Sustainability and Banking

I confirm that I have read this submission and believe that I have an appropriate level of expertise to confirm that it is of an acceptable scientific standard.

# Reviewer report: Reviewer response for version 1

**DOI:** 10.5256/f1000research.169036.r317726

**Published:** 2024-09-12

**Authors:** Madhusmita Mohanty

**Affiliations:** 1 VIT-AP School of Business,, VIT-AP University Amaravati, Amaravati, Andhra Pradesh, India

The article title, “Incentivizing green building technology: A financial perspective on sustainable development in India” provides comprehensive knowledge about financial incentives for green building at various levels. The research presented with latest literature reviews. Authors explore central as well as state government supported for green building. The authors covered all aspects in a clearly and deliberately manner.

 It is a nice research work. Authors have well-articulated.

Is the review written in accessible language?

Yes

Are all factual statements correct and adequately supported by citations?

Yes

Are the conclusions drawn appropriate in the context of the current research literature?

Yes

Is the topic of the review discussed comprehensively in the context of the current literature?

Yes

Reviewer Expertise:

Finance, Banking

I confirm that I have read this submission and believe that I have an appropriate level of expertise to confirm that it is of an acceptable scientific standard.

# Reviewer report: Reviewer response for version 1

**DOI:** 10.5256/f1000research.169036.r317730

**Published:** 2024-09-10

**Authors:** Mohammed Ismail Iqbal

**Affiliations:** 1 College of Engineering and Technology,, University of technology and sciences, Nizwa,, Oman

The Paper title  “Incentivizing green building technology: A financial perspective on sustainable development in India”  is interesting and author emphasises green building   development incentive. Author tried to cover all aspect of green building aspect related to financial benefit. There are some improvement required which described below:

 1.  Introduction section required more elaborate related to background of green building incentive, challenges, motivation of research etc.

 2.  Literature review section   required updated literature related  to green  building  incentive.  Grammatical mistake in figure  1( Reference).

 3. Methodology of the research can  be  presented though   a  flow  chart  for   convenience  of  readers.

 4.  Author mixed conclusion and recommendation in  section 7 and Recommendation shall described in  separated  section.

Is the review written in accessible language?

Yes

Are all factual statements correct and adequately supported by citations?

Yes

Are the conclusions drawn appropriate in the context of the current research literature?

Yes

Is the topic of the review discussed comprehensively in the context of the current literature?

Yes

Reviewer Expertise:

Environmental Engineering, Sustainability,......

I confirm that I have read this submission and believe that I have an appropriate level of expertise to confirm that it is of an acceptable scientific standard.

# Response

**Published:** 2024-09-10

**Authors:** Rakesh Kumar

**Affiliations:** Management, Uttaranchal University, Dehradun, Uttarakhand, India

Dear  Reviewer,

 Thank  you  for  your comments. Kindly  see responses:-

 1.  Introduction section required more elaborate related to   background of green  building   incentive,  challenges, motivation  of research  etc.

**Response of comment 1 : ** Thank  you  for  your  comments.   We have added Research  motivation in the introduction section  as described  follows:-

 1.1 Research Motivation 

 This research review on green building   incentive in India.  A life cycle cost method results from the long-term advantages of green building over conventional construction. A flexible design, such incremental tax rebates, can motivate stakeholders to construct and run environmentally friendly businesses. This method lowers the initial cost of green building projects and promotes sustainable habits more successfully. The study focuses on  existing   green  financial  incentive given by  central  government   and  state  government  separately. The policymakers can make an analysis in terms of cost, environmental clearance, and easy access for the construction of green buildings. The study explores the long-term benefits for consumers of the adoption of green building projects.

 2.   Literature review section   required updated literature related to green building incentive.  Grammatical mistake in figure 1(Reference).

**Response of comment 2:  **Thank you  for  your  comments.    The  objective  of   study  support literature  reviews.  We have added    more six   recent  literature  which  support  objective  of   research , as per your   suggestion.  Figure 1 has  been  updated.  The  changes  made in  manuscripts are as follows:-

 Green building is a sustainable substitute for conventional building that has less of an impact on the environment and climate change[51]. To make the finished product sustainable, green building technologies (GBTs) like solar power, prefabricated concrete, and green roof technology must be used. GBTs have a lot to offer in terms of sustainability, like lower energy use and better water efficiency. But there are a lot of obstacles in the way of GBT adoption in the construction industry. Although a lot of research has been done on GBT adoption difficulties, little is known about GBT adoption barriers in underdeveloped nations[52]. Developing solutions to overcome these limitations, particularly in developing nations like Ghana, requires a deeper understanding of them. Effective promotion requires understanding the criticalities of GBT adoption barriers at different stages[49].

 India must recycle trash due to rising environmental concerns, economic expansion, and population growth. Due to the country's growing urbanisation, the building industry in India produces 10–12 million tonnes of garbage annually. The Indian green building movement has been greatly aided by the establishment of the Indian Green Building Council (IGBC), which was founded by US President Bill Clinton. The Indian Northeastern Mountains encounter a variety of special difficulties, such as rockfall, landslides, debris flow, slope sinking, and erosion of riverbanks. In Northeast India, the IGBC has established a green building footprint of more than 30 million square feet[50].

 In four Asian nations—China, India, Indonesia, and Japan—policymakers are pushing energy efficiency and renewable energy due to increased awareness of climate change. The study looks at market-based tools and regulations that provide incentives to lower energy intensity

 References

 49.       Chan, A. P. C., Darko, A., Olanipekun, A. O., & Ameyaw, E. E. (2018). Critical barriers to green building technologies adoption in developing countries: The case of Ghana. *Journal of cleaner production*, *172*, 1067-1079.

 50        Jain, M. S., & Kakati, J. (2024). A statistical approach to evaluate the effect of obstacles on green building development in Northeast India. World Development Sustainability, 4, 100119.

 51        Potluri, S., & Phani, B. V. (2020). Incentivizing green entrepreneurship: A proposed policy prescription (a study of entrepreneurial insights from an emerging economy perspective). *Journal of Cleaner Production*, *259*, 120843.

 52        Shazmin, S. A. A., Sipan, I., Sapri, M., Ali, H. M., & Raji, F. (2017). Property tax assessment incentive for green building: Energy saving based-model. *Energy*, *122*, 329-339.

 53        Sarker, T., Taghizadeh-Hesary, F., Mortha, A., & Saha, A. (2021). The Role of Fiscal Incentives and Market-Based Incentives in Promoting Energy Efficiency in the Industrial Sector: Case Studies from Asia. *Energy Efficiency Financing and Market-Based Instruments*, 131-149.

 3. Methodology of the research can be presented though   a flow chart  for   convenience  of  readers.

**Response of comment 3: ** Thank you for your  comments.    Flow chart inserted  in   methodology  section

 4.  Author mixed conclusion and recommendation in section 7.   Recommendation shall be described in separated section.

**Response of comment 4 :**  Thank you for your  comments.  We have updated recommendation and conclusion  section  in manuscript.

# Reviewer report: Reviewer response for version 1

**DOI:** 10.5256/f1000research.169036.r317724

**Published:** 2024-09-06

**Authors:** Vinay Kandpal

**Affiliations:** 1 Graphic Era Deemed to be University, Dehradun, Uttarakhand, India

Nice work done. Some suggestions that could enhance the quality of paper:

 1. Research motivation describes what motivates researchers and what they would like to achieve as a result of their studies. Therefore, the author can add a paragraph on research motivation in the introduction.

 2. The author has written a research gap in the introduction section. But the same can be explained in detail with supportive relevant recent literature published recently. 

 3. Authors could discuss the scope for future work.

 4. Literature Review should be extensive and include more supporting literature published in the last 10 years.

Is the review written in accessible language?

Yes

Are all factual statements correct and adequately supported by citations?

Yes

Are the conclusions drawn appropriate in the context of the current research literature?

Yes

Is the topic of the review discussed comprehensively in the context of the current literature?

Yes

Reviewer Expertise:

Sustainability and Banking

I confirm that I have read this submission and believe that I have an appropriate level of expertise to confirm that it is of an acceptable scientific standard, however I have significant reservations, as outlined above.

# Response

**Published:** 2024-09-09

**Authors:** Rakesh Kumar

**Affiliations:** Management, Uttaranchal University, Dehradun, Uttarakhand, India

Dear  Reviewer,

 Thank  you  for your  comments.  Kindly find  the response of  your  Comments 

**Response Sheet**

**1. Research motivation describes what motivates researchers and what they would like to achieve because of their studies. Therefore, the author can add a paragraph on research motivation in the introduction**.

 Response of comment 1 :  Thank  you  for  your  comments.   We have  added  Research  motivation in the introduction section  as described  follows:-

 1.1 Research Motivation 

 This research review on green building   incentive in India.  A life cycle cost method results from the long-term advantages of green building over conventional construction. A flexible design, such incremental tax rebates, can motivate stakeholders to construct and run environmentally friendly businesses. This method lowers the initial cost of green building projects and promotes sustainable habits more successfully. The study focuses on  existing   green  financial  incentive given by  central  government   and  state  government  separately. The policymakers can make an analysis in terms of cost, environmental clearance, and easy access for the construction of green buildings. The study explores the long-term benefits for consumers of the adoption of green building projects.

**2. The author has written a research gap in the introduction section. But the same can be explained in detail with supportive relevant recent literature published recently.**

 Response of comment 2:  Thank  you  for  your  comments.    The  objective  of   study  support literature  reviews.  We have added    more  six   recent  literature  which  support  objective  of   research , as per your   suggestion. The  changes  made in  manuscripts are as follows:-

 Green building is a sustainable substitute for conventional building that has less of an impact on the environment and climate change[51]. To make the finished product sustainable, green building technologies (GBTs) like solar power, prefabricated concrete, and green roof technology must be used. GBTs have a lot to offer in terms of sustainability, like lower energy use and better water efficiency. But there are a lot of obstacles in the way of GBT adoption in the construction industry. Although a lot of research has been done on GBT adoption difficulties, little is known about GBT adoption barriers in underdeveloped nations[52]. Developing solutions to overcome these limitations, particularly in developing nations like Ghana, requires a deeper understanding of them. Effective promotion requires understanding the criticalities of GBT adoption barriers at different stages[49].

 India must recycle trash due to rising environmental concerns, economic expansion, and population growth. Due to the country's growing urbanisation, the building industry in India produces 10–12 million tonnes of garbage annually. The Indian green building movement has been greatly aided by the establishment of the Indian Green Building Council (IGBC), which was founded by US President Bill Clinton. The Indian Northeastern Mountains encounter a variety of special difficulties, such as rockfall, landslides, debris flow, slope sinking, and erosion of riverbanks. In Northeast India, the IGBC has established a green building footprint of more than 30 million square feet[50].

 In four Asian nations—China, India, Indonesia, and Japan—policymakers are pushing energy efficiency and renewable energy due to increased awareness of climate change. The study looks at market-based tools and regulations that provide incentives to lower energy intensity

 References

 49.       Chan, A. P. C., Darko, A., Olanipekun, A. O., & Ameyaw, E. E. (2018). Critical barriers to green building technologies adoption in developing countries: The case of Ghana. *Journal of cleaner production*, *172*, 1067-1079.

 50        Jain, M. S., & Kakati, J. (2024). A statistical approach to evaluate the effect of obstacles on green building development in Northeast India. World Development Sustainability, 4, 100119.

 51        Potluri, S., & Phani, B. V. (2020). Incentivizing green entrepreneurship: A proposed policy prescription (a study of entrepreneurial insights from an emerging economy perspective). *Journal of Cleaner Production*, *259*, 120843.

 52        Shazmin, S. A. A., Sipan, I., Sapri, M., Ali, H. M., & Raji, F. (2017). Property tax assessment incentive for green building: Energy saving based-model. *Energy*, *122*, 329-339.

 53        Sarker, T., Taghizadeh-Hesary, F., Mortha, A., & Saha, A. (2021). The Role of Fiscal Incentives and Market-Based Incentives in Promoting Energy Efficiency in the Industrial Sector: Case Studies from Asia. *Energy Efficiency Financing and Market-Based Instruments*, 131-149.

**3. Authors could discuss the scope for future work.**

 Response of comment 3:  Thank  you  for  your  comments. We have added scope of future work. The changes  made in  manuscripts are as follows:-

 9. Scope of Future  Work

 This research finds out different financial incentives for green building technology. The research focusses on existing market-based instruments available for green building technology and their benefits to users. The study explores incentives given by the central and state governments for green building projects. This study reviews the financial benefits within the Indian region. However, future research may be conducted in other regions. There is scope for comparison of green building incentives between two regions. The further study may be conducted on consumer perspective-related challenges faced to adoption of green buildings.

**4. Literature Review should be extensive and include more supporting literature published in the last 10 years.**

 Response of comment 4:  Thank you  for  your  comments.     We have added    more six   recent literature which  support  research , as per your   suggestion. The changes made in  manuscripts are as follows:-

 Green building is a sustainable substitute for conventional building that has less of an impact on the environment and climate change[51]. To make the finished product sustainable, green building technologies (GBTs) like solar power, prefabricated concrete, and green roof technology must be used. GBTs have a lot to offer in terms of sustainability, like lower energy use and better water efficiency. But there are a lot of obstacles in the way of GBT adoption in the construction industry. Although a lot of research has been done on GBT adoption difficulties, little is known about GBT adoption barriers in underdeveloped nations[52]. Developing solutions to overcome these limitations, particularly in developing nations like Ghana, requires a deeper understanding of them. Effective promotion requires understanding the criticalities of GBT adoption barriers at different stages[49].

 India must recycle trash due to rising environmental concerns, economic expansion, and population growth. Due to the country's growing urbanisation, the building industry in India produces 10–12 million tonnes of garbage annually. The Indian green building movement has been greatly aided by the establishment of the Indian Green Building Council (IGBC), which was founded by US President Bill Clinton. The Indian Northeastern Mountains encounter a variety of special difficulties, such as rockfall, landslides, debris flow, slope sinking, and erosion of riverbanks. In Northeast India, the IGBC has established a green building footprint of more than 30 million square feet[50].

 In four Asian nations—China, India, Indonesia, and Japan—policymakers are pushing energy efficiency and renewable energy due to increased awareness of climate change. The study looks at market-based tools and regulations that provide incentives to lower energy intensity

 References

 49.       Chan, A. P. C., Darko, A., Olanipekun, A. O., & Ameyaw, E. E. (2018). Critical barriers to green building technologies adoption in developing countries: The case of Ghana. *Journal of cleaner production*, *172*, 1067-1079.

 50        Jain, M. S., & Kakati, J. (2024). A statistical approach to evaluate the effect of obstacles on green building development in Northeast India. World Development Sustainability, 4, 100119.

 51        Potluri, S., & Phani, B. V. (2020). Incentivizing green entrepreneurship: A proposed policy prescription (a study of entrepreneurial insights from an emerging economy perspective). *Journal of Cleaner Production*, *259*, 120843.

 52        Shazmin, S. A. A., Sipan, I., Sapri, M., Ali, H. M., & Raji, F. (2017). Property tax assessment incentive for green building: Energy saving based-model. *Energy*, *122*, 329-339.

 53        Sarker, T., Taghizadeh-Hesary, F., Mortha, A., & Saha, A. (2021). The Role of Fiscal Incentives and Market-Based Incentives in Promoting Energy Efficiency in the Industrial Sector: Case Studies from Asia. *Energy Efficiency Financing and Market-Based Instruments*, 131-149.
